# Drills or Distress? Understanding the Psychological Effects of Active Shooter Drills on Adolescents

**DOI:** 10.1177/10598405251369548

**Published:** 2025-09-08

**Authors:** Jeff R. Temple, Elizabeth Baumler, Christian Paige Owen, Leila Wood

**Affiliations:** 1 School of Behavioral Health Sciences, 12338 University of Texas Houston Health Science Center, Houston, TX, USA; 2 Cizik School of Nursing, 16171 University of Texas Health Science Center at Houston, Houston, TX, USA

**Keywords:** active shooter drills, adverse childhood experiences, anxiety, suicide, disability

## Abstract

**Background:** We sought to better understand the impact of exposure to active shooter drills (ASDs) on students’ perceived stress, including those with disabilities and prior experiences with trauma. **Methods:** We used data from a longitudinal study of ethnically diverse students (n = 2,033; 53.5% female) originally recruited in 2018 and followed annually thereafter. In addition to self-reporting on their perceived stress with ASDs, participants responded to questions about adverse childhood experiences, anxiety, suicidal ideation, and disability. **Results:** Female students and those with a disability, probable anxiety, and suicidal ideation were more likely to be anxious after an ASD. **Conclusion:** While necessary given the increasing reality of school shootings in the United States, our results indicate that active shooter drills should be implemented with concern for vulnerable groups, including distressed students and those with a history of traumatic experiences or who have a disability.

## Introduction

While accounting for a small portion of annual gun violence deaths, school shootings are an increasingly frightening reality in the United States. Indeed, there were 51, 38, and 39 school shootings resulting in injury or death from 2022 and 2024, respectively (Education Week, 2024). Given the alarming frequency and devastating impact of these shootings on school communities, a vast majority of states require schools to have lockdown or active shooting drills (ASDs) to protect students and staff (Churchill & Kriel, 2024; National Center for Education Statistics, 2024). These drills generally involve practicing procedures like turning off lights, keeping silent, and locking doors, and can vary from low-sensorial (e.g., tabletop exercises that do not involve students) to high sensorial (e.g., role-play exercises with a “shooter”). Opinions vary regarding the benefit and application of ASDs, and the evidence-base to support their effectiveness in mitigating the consequences of school shootings is lacking (Miotto & Cogan, 2023). Moreover, research is mixed on whether ASDs result in unintended negative consequences, including increased psychological distress (Huskey & Connell, 2021; Schildkraut & Nickerson, 2022)—especially among vulnerable students.

The National Association of School Nurses (NASN) identifies student wellbeing as a research priority (NASN, 2025), emphasized by school nurses’ reported need for more training to support students who experience adverse childhood experiences (ACEs) and mental health issues (Morse et al., 2022). As schools attempt to strengthen and standardize emergency management plans in response to violence, school nurses are being called upon as key leaders due to their scope and responsibility to identify and promptly mitigate risks that interfere with the safety, health, and wellbeing of staff and students (DeVos et al., 2018; NASN, 2024; Selekman & Melvin, 2017). As part of a multidisciplinary team, school nurses are well positioned to incorporate the needs of students as they engage in planning, implementing, and evaluating safety trainings aimed at preventing and improving the response to health threats and violent events, including school shootings (Idowu, 2023; Maughan et al., 2018). The potential consequences of ASDs on student mental health warrant further study to clarify implications for school nurses and administrators. In this brief research report, we begin to address this gap in knowledge by assessing self-reported experiences with and perceived stress of exposure to active shooter drills within the context of prior ACEs, psychological distress, and disability status.

## Method

The sample included an ethnically diverse group of students participating in an ongoing randomized controlled trial examining the effectiveness of a healthy relationships program (Temple et al., 2021, 2025). In 2018, schools from two large urban/exurban school districts were randomized to either intervention (n = 12) or control (n = 12) and then grade 7 students completed the baseline survey prior to receiving the intervention (i.e., *Fourth R*) or standard health curricula (year 1). *Fourth R* is an interactive classroom-based healthy relationship curriculum designed to reduce dating violence and other risky behaviors. The program is delivered by existing teachers and consists of 21 lessons on injury prevention, substance use, and human growth and development. While all students in the intervention schools received *Fourth R*, only those who received parental permission and provided assent completed surveys at baseline (prior to intervention) and annually thereafter. We analyzed data provided from students in year 6 of the study (n = 2,033; retention = 71% of baseline participants), which is when we inquired about the relevant variables. These data were collected in 2024. Participants had just graduated (mean age = 18.7, SD = 0.82) and were reporting retrospectively about their time in high school. Participants (53.5% female) were Black (23.4%), White (6.1%), Hispanic (36.5%), Asian (16.0%), and multi-ethnicity/other (17.9%).

We measured exposure and perceived reaction to ASDs with two items (i.e., “How many times did your high school have active shooter drills?” and “How did you typically feel after the active shooter drill?”). We used the DSM-5 Level 1 Cross-Cutting Symptom Measure (American Psychiatric Association, 2013) to assess suicidal ideation (1 yes/no item; i.e., “Have you ever thought about killing yourself?”) and anxiety (3 items anchored by not at all [0] and nearly every day [4]; i.e., During the past two weeks…“How often have you felt nervous, anxious, or scared”; “How often have you not been able to do things you wanted to or should have done because they made you nervous?”; and How often have you not been able to stop worrying?”). Participants who endorsed a 3 (more than half the days) or more on any of the items were considered probable for anxiety. We used the Philadelphia ACEs to tally the number of conventional (e.g., child maltreatment) and expanded (e.g., racism and neighborhood safety) forms of childhood trauma experienced by participants (Cronholm et al., 2015). Lastly, we adapted questions from the National Study of Student Engagement (Kuh, 2001) to assess the presence of a range of physical, cognitive, psychiatric, sensory, and developmental disabilities (e.g., mobility impairment, Attention Deficit Hyperactivity Disorder, and autism) and asked students to mark all that apply.

The first author's institutional review board approved the study. We obtained active parent/guardian consent and student assent prior to administering the surveys. Students received a $30 gift card for completing the survey. We conducted descriptive analysis and chi square for association of variables with ASD. Statistical analysis was performed using Stata statistical software (Stata/IC 15.1; Stata Corp, College Station, TX).

## Results

Nearly three-quarters of participants (73.4%) experienced an ASD while in high school (17% reported having less than one ASD per year, 29% reported about one per year, and 28% reported more than one per year). For analyses, we excluded students who reported never experiencing an ASD during high school (n = 530; 26.6% of the sample). Of those who reported engaging in at least one ASD in the previous year, 9.9% reported a disability, 30.9% had probable anxiety, 36.3% had lifetime suicidal ideation, and 82.5% had at least one ACE (34.6% had 1–2; 33.4% had 3–5, and 14.5% had 6+). As shown in Table 1, female students and those with a disability, probable anxiety, and history of suicidal ideation were more likely to endorse feeling anxious after an ASD. Further, we observed a linear positive association between number of ACEs and feeling anxious after an ASD. Frequency of ASDs were inversely linked to anxiety.

**Table 1.** Reaction to Active Shooter Drills by Demographics, Disability Status, Psychological Distress, and Prior Trauma.

		Did not impact		Felt anxious or scared		Felt safe or relieved		Test of association across strata	
	n	x	%	x	%	x	%	Chi-square, df^b^	*p* Value^c^
Overall	1,465^a^	1044	71.4	261	17.9	157	10.7		
Female	810	522	64.4	210	25.9	78	9.6	80.51, 2	<.001
Male	651	521	80.0	51	7.8	79	12.1		
Probable anxiety	451	294	65.2	112	24.8	45	10.0	21.53, 2	<.001
No anxiety	1009	748	74.1	149	14.8	112	11.1		
Suicidal ideation	526	387	73.6	117	22.2	22	4.2	41.81, 2	<.001
No suicidal ideation	924	648	70.1	143	15.5	133	14.4		
Disability	142	93	65.5	40	28.2	9	6.3	12.80, 2	.002
No disability	1295	935	72.2	217	16.8	143	11.0		
No ACEs	256	196	76.6	30	11.7	30	11.7	30.45, 6	<.001
1–2 ACEs	506	368	72.7	76	15.0	62	12.3		
3–5 ACEs	487	351	72.1	94	19.3	42	8.6		
6 or more ACEs	212	128	60.4	61	28.8	23	10.8		
< 1 Time year	335	222	66.3	78	23.3	35	10.4	15.9, 4	.003
About 1 time a year	581	440	75.7	91	15.7	50	8.6		
> 1 Time a year	546	382	70.0	92	16.8	72	13.2		

a Subgroup defined by excluding those reporting “never” to high school (HS) conducting active shooter drills.

b Pearson Chi-square test of association, degrees of freedom.

c *p* Values not adjusted for multiple comparison.

ACE = adverse childhood experience.

## Discussion

Given the reality of school shootings and the need to ensure school safety, lockdown or active shooter drills are likely a fact of life in the United States. Consistent with recommendations from the American Academy of Pediatrics (Schonfeld et al., 2020), our findings suggest that these drills should be implemented with concern for vulnerable groups of students, including those with mental health problems, traumatic experiences, and disability. These findings are consistent with research showing that individuals with psychological distress are at increased risk for future stressors (Breslau et al., 2008). Specifically, it is likely that students with early life adversity and those with more depression and anxiety are particularly vulnerable to stressful experiences (in this case, ASDs and fear of active shooters) (Bandoli et al., 2017).

Implications for school nurses and administrators include ongoing trauma-informed assessment, planning, and collaboration as members of the emergency preparedness team to identify and facilitate mental health support for students at risk of psychological distress during ASDs. Additionally, instrumental and structural adaptations for students with disabilities must be considered, as these students may be limited in their ability to comply with ASD instructions (e.g., lockdown, counter/resist, and evacuate) thus heightening their sense of vulnerability about an actual event. Because school nurses have insight into the medical needs of all students, they are well-positioned to promote awareness of gaps in planning for ASDs and take the lead in developing procedures to be enacted by all students and staff (McIntosh et al., 2020).

Limitations include the cross-sectional design, limited measures of suicidal ideation and experiences with ASDs, and the retrospective self-reporting of participants’ experiences and perceptions. With respect to the latter point, we cannot determine if anxious students (or those with suicidality or increasing ACEs) were indeed more anxious postactive shooter drills or if they simply remember them as more anxiety provoking. Similarly, we did not assess when and how many times through high school that participants were exposed to ASDs. Regardless, their experiences appear to be related to negative outcomes, and these results provide a foundation from which to build more comprehensive studies to further explore this critically important issue. For example, future longitudinal research should assess predrill and postdrill distress, as well as detailed school-level information about when and how the drills were conducted (e.g., trauma informed and high sensorial).

Considering these limitations, as well as study strengths (e.g., large ethnically diverse sample), findings highlight the importance of potential unintended consequences of ASDs on students. To mitigate these negative impacts, we suggest that ASDs are (1) scheduled in advance to reduce the stress of uncertainty and allow for school staff (i.e., teachers, counselors, and nurses) to prepare vulnerable students; (2) debrief students and provide counseling resources postdrill; (3) administered only after accommodations are made for students with disabilities; and (4) limited to tabletop exercises, involve only school staff, or restricted to low-sensorial activities.

## Conclusion

Schools play a critical role in fostering safe environments for students to thrive physically and academically, which drives the continued requirement and implementation of ASDs. However, our findings suggest that ASDs may have unintended and negative mental health consequences on students. Design and planning of ASDs should include expertise from school nurses and extend beyond physical safety concerns to also include considerations for mental health consequences, particularly for students at increased risk due to disability or history of adverse experiences.
